# A new chymotrypsin-like serine protease involved in dietary protein digestion in a primitive animal, *Scorpio maurus*: purification and biochemical characterization

**DOI:** 10.1186/1476-511X-10-121

**Published:** 2011-07-21

**Authors:** Hanen Louati, Nacim Zouari, Nabil Miled, Youssef Gargouri

**Affiliations:** 1 Laboratoire de Biochimie et de Génie Enzymatique des Lipases, ENIS, Université de Sfax, route de Soukra 3038, BP 1173 Sfax-Tunisia

## Abstract

**Background:**

Most recent works on chymotrypsins have been focused on marine animals and insects. However, no study was reported in chelicerate.

**Results:**

Scorpion chymotrypsin-like protease (SCP) was purified to homogeneity from delipidated hepatopancreases. The protease NH_2_-terminal sequence exhibited more than 60% monoacids identity with those of insect putative peptidases. The protease displayed no sequence homology with classical proteases. From this point of view, the protease recalls the case of the scorpion lipase which displayed no sequence homology with known lipases. The scorpion amylase purified and characterized by our time, has an amino-acids sequence similar to those of mammalian amylases. The enzyme was characterized with respect its biochemical properties: it was active on a chymotrypsin substrate and had an apparent molecular mass of 25 kDa, like the classically known chymotrypsins. The dependence of the SCP activity and stability on pH and temperature was similar to that of mammalian chymotrypsin proteases. However, the SCP displayed a lower specific activity and a boarder pH activity range (from 6 to 9).

**Conclusion:**

lower animal have a less evaluated digestive organ: a hepatopancreas, whereas, higher ones possess individualized pancreas and liver. A new chymotrypsin-like protease was purified for the first time from the scorpion hepatopancreas. Its biochemical characterization showed new features as compared to classical chymotrypsin-higher-animals proteases.

## Background

Proteases, including trypsin and chymotrypsin, constitute one of the largest families of enzymes in the animal kingdom involved in dietary protein digestion [1]. Trypsin and trypsin-like proteases have received a great interest and were well characterized. However, informations about chymotrypsins are less available [2]. Chymotrypsins cleave the peptides on the carboxyl side of phenylalanine, tyrosine and tryptophan residues and have been purified and characterized from mammals [3,4]; fishes [5-7] and crustaceans such as scallop (*Pecten maximus*) [8] and shrimp (*Penaus vannamei*) [9]. Chymotrypsins act primarily as an aid of digestion and as anti-inflammatory agent by preventing tissue damage and fibrin clots. Consequently, they were used for treating bacterial, viral, fungal, and parasitic infections in mammals. Chymotrypsins were shown to possess an anti-cell-cell adhesion activity [10]. Most recent works on chymotrypsins have been focused on marine animals, while studies on chymotrypsins from terrestrial arthropods are scare. Scorpion, one of the most ancient chelicerates, was chosen in this work as a model of a primitive animal to characterize the enzymes involved in dietary proteins digestion.

Scorpions have changed little since the Silurian (450 million years), and were considered as the oldest known terrestrial species. The food of scorpions is composed exclusively of a live arthropods, insects, myriapods. It was well known that scorpions could survive several weeks without food or water. The scorpion digestive glands, which represent our starting tissue in this work, occupy most of the space in the preabdomen and are conspicuous, clumped together and cannot be distinguished as separate glands. The scorpion digestive glands were studied at the ultrastructure level [11,12]. The digestive diverticula of scorpion were composed of two differentiated cells: basophilic cells and digestive cells. Whereas basophilic cells produce exoenzymes, digestive cells ensure intracellular digestion of nutrients absorbed by pinocytosis and store lipids, glycogen and mineral salts. The digestive mode of the scorpion associates a primitive intracellular process with an advanced extracellular one [11,12]. A lipase and an amylase were previously purified and characterized from digestive glands of the scorpion [13,14]. The scorpion digestive lipase was shown to possess new biochemical and structural properties in comparison to higher animals'digestive lipases. Furthermore, this enzyme was immunocytolocalized in the digestive cells and was thought to be persensible for an intracellular digestive process [12]. In contrast to what has been observed for scorpion lipase, the scorpion digestive amylase (SDA) was shown to share an NH_2_-terminal sequence similarity with pancreatic amylases. Despite the primitive character of the scorpion, similar biochemical properties have been observed between SDA and known pancreatic amylases. However, the absence of cross-immunoreactivity between porcine pancreatic amylase and anti-SDA serum strengthens the idea that SDA could be structurally different from mammalian pancreatic amylases [14].

To the best of our knowledge, no proteases from the scorpion digestive glands have been purified and characterized.

This paper reports, for the first time, the purification to homogeneity of an active chymotrypsin-like protease from the scorpion digestive glands. This protease was characterized with respect to its biochemical properties.

## Materials and methods

### Reagents

Casein sodium salt from bovine milk, ethylenediaminetetraacetic acid (EDTA), phenylmethylsulfonyl fluoride (PMSF), dimethylsulfoxide (DMSO), dithio-bis-nitrobenzoic acid (DTNB), trichloroacetic acid (TCA), glycine, ammonium sulphate, β-mercaptoethanol, benzoyl-Arg-*p*-nitroanilide (BAPNA), N-Succinyl-L-Ala-L-Ala-L-Pro-L-Phe-p-nitroanilide (SAPNA), N-alpha-(p-toluene sulfonyl)-L-arginine methyl ester (TAME), and protein markers for molecular masses 14,200-45,000 Da were purchased from Sigma Chemical Co. (USA). Tris (hydroxymethyl) aminomethane was procured from Panreac Quimica SA (Spain). Sodium dodecyl sulphate (SDS), acrylamide, ammonium persulphate, N, N, N', N'-tetramethyl ethylenediamine (TEMED), and Coomassie Brilliant Blue R-250 were from Bio-Rad Laboratories (Mexico). DEAE-Sephadex, Sephadex G-100, Sephadex G-50 and SP-Sepharose were from Pharmacia Biotech (Sweden). All other reagents were of analytical grade.

### Proteins

Pure porcine trypsin protease (PTP), pure bovine chymotrypsin protease (BCP) and bovine serum albumin (BSA) were purchased from Sigma Chemical Co. (USA).

### Determination of protein concentration

Protein concentration was determined as described previously [15], using BSA (E^1%^_1 cm _= 6.7) as reference.

### Animals

Scorpions (chelicerate, scorpionidae, *Scorpio maurus*) were collected alive from the area of Agareb (Sfax, Tunisia) and frozen until death.

### Delipidation of scorpion hepatopancreases

After defreezing, preabdomens were cleared from the cuticle and delipidated according to the method described previously [16]. After delipidation, 15 g of powder were obtained from 60 g of fresh tissue isolated from 200 scorpions.

### Proteolytic activities

In order to ensure linear course of reaction, preliminary enzyme assays were performed in order to determine the concentration of substrate, the volume of crude enzyme extract and the time course of the reaction (data not shown). Protease activity was measured by the method of Kembhavi using casein as substrate [17]. Enzyme solution (0.5 mL) suitably diluted was mixed with 0.5 mL of 100 mM Tris-HCl pH 9, containing 1% casein, and incubated for 10 min at 50°C. The reaction was stopped by addition of 0.5 mL TCA (20%, w/v). The mixture was allowed to stand at room temperature for 15 min and then centrifuged at 13,000 rpm for 15 min to remove the precipitate. The absorbance was measured at 280 nm. A standard curve was generated using solutions of 0-50 mg/mL tyrosine. One unit of protease activity was defined as the amount of enzyme, which liberated 1 μg tyrosine in 1 min at 50°C and at pH 9 for scorpion chymotrypsin-like protease (SCP) and at pH 7 for porcine trypsin protease (PTP) and bovine chymotrypsin protease (BCP).

Chymotrypsin activity was determined using 20 μM N-Succinyl-L-Ala-L-Ala-L-Pro-L-Phe-p-nitroanilide (SAPNA) in 10 mM CaCl_2_, 50 mM Tris-HCl buffer, pH 7.5. Then, 20 μL of the enzymatic extracts was added to 980 μL of the substrate solution and, after incubation at 25°C, the absorbance at 410 nm was recorded [18]. Chymotrypsin activity was expressed as U/mg. Values of different enzymatic activities are the means of three independent experiments.

Trypsin activity was determined using benzoyl-Arg-*p*-nitroanilide (BAPNA) as substrate [19]. BAPNA (1 mM) was dissolved in 1 mL of DMSO and then diluted to 100 mL with 10 mM CaCl_2_, 50 mM Tris-HCl buffer, pH 7.5. Then, 25 μL of the enzymatic extracts were added to 1.25 mL of BAPNA at 25°C and the absorbance at 410 nm was recorded after 5 min. Trypsin activity was expressed as U/mg, were U was to the amount of enzyme yielding 0.1 unit of absorbance per min.

Esterase activity of trypsin was determined using N-alpha-(p-toluene sulfonyl)-L-arginine methyl ester (TAME) as the substrate on the basis of the method of Hummel with slight modifications [20]. The reaction mixture comprised 2.8 mL of the substrate (1 mM TAME in 0.05 M Tris-HCl buffer, pH 8.5) and 0.2 mL of the enzyme solution. The reaction mixture was thoroughly mixed, and the release of toluene sulfonyl arginine was measured at 247 nm.

### Effect of protease inhibitors

The effects of enzyme inhibitors on chymotrypsin activity were studied using phenylmethylsulfonyl fluoride (PMSF), thiol reagent dithio-bis-nitrobenzoic acid (DTNB), benzamidine, β-mercaptoethanol and ethylene-diaminetetraacetic acid (EDTA). The purified enzyme was preincubated with inhibitors for 30 min at 25°C and then, the remaining enzyme activity was estimated using casein as a substrate. The enzyme activity measured in the absence of inhibitors was taken as 100%.

### Purification steps of SCP

Delipidated powder (10 g) of scorpion hepatopancreases was suspended in 100 mL of buffer A (10 mM Tris-HCl pH 8, 10 mM NaCl and 4 mM CaCl_2_). The mixture was stirred during 30 min at 4°C, then centrifuged for 30 min at 12,000 rpm.

**- DEAE-Sephadex: **The supernatant was loaded onto a DEAE-Sephadex column (3 × 20 cm) equilibrated with buffer A. Under these conditions, the enzyme did not adsorb on the cationic support and was eluted during a washing step by the same buffer A. This step was important to eliminate many contaminants and to clarify the enzymatic solution (data not shown).

**- Ammonium sulfate precipitation: **The pooled fractions of DEAE-Sephadex column were subjected to ammonium sulfate precipitation. SCP precipitated in a saturation of 75% ammonium sulfate added under stirring conditions at 4°C. After centrifugation (30 min at 12,000 rpm), the pellet was resuspended in 12 mL of buffer B (10 mM sodium acetate pH 6, 10 mM NaCl and 4 mM CaCl_2_). Insoluble material was removed by centrifugation for 5 min at 12,000 rpm.

**Figure 1:**
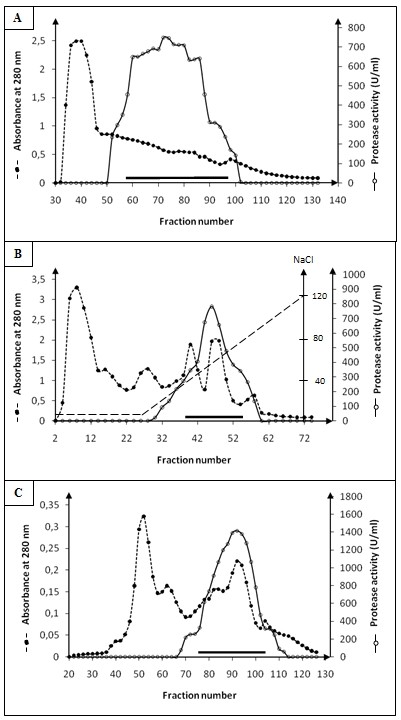
**Chromatography of SCP on Sephadex G-100 and FPLC SP-Sepharose**. (A) Chromatography of SCP on Sephadex G-100. The column (3.2 × 100 cm) was equilibrated with buffer A (10 mM acetate pH 6, 10 mM NaCl and 4 mM CaCl_2_). The elution of protease was performed with the same buffer at a rate of 30 mL/h and 5.6 mL by fraction. (B) Chromatography of SCP on FPLC SP-Sepharose. The column was equilibrated with buffer A (10 mM Tris-HCl pH 8, 10 mM NaCl and 4 mM CaCl_2_); a linear gradient was applied from 10 to 120 mM NaCl in buffer B; the elution of protease was performed with the same buffer at a rate of 2 mL/min and 3 mL by fraction. **C: **Chromatography of SCP on Sephadex G-50. The column (3 × 90 cm) was equilibrated with buffer A. The elution of protease was performed with the same buffer at a rate of 30 mL/h and 4.8 mL by fraction. SCP activity was measured as described in materials and methods section using casein as substrate. SCP activity was measured as described in materials and methods section using casein as substrate.

**- Filtration on Sephadex G-100: **The supernatant issued from ammonium sulfate precipitation, was loaded on a gel filtration Sephadex G-100 column (3.2 × 100 cm) equilibrated with buffer B. Elution of protease was performed with the same buffer at 30 mL/h. The fractions containing the protease activity (eluted at 1.4 void volume) were pooled (fractions from 58 to 98) (Figure 1A).

**- FPLC Anion exchange SP- Sepharose: **The pooled fractions of Sephadex G-100 column were concentrated and injected onto an FPLC SP-Sepharose column equilibrated with buffer B. The column (1.6 × 15 cm) was rinsed with the same buffer. Then, proteins were eluted with a linear gradient of NaCl prepared in buffer B. SCP was eluted at a salt concentration of 40-80 mM NaCl (Figure 1B).

**Figure 2:**
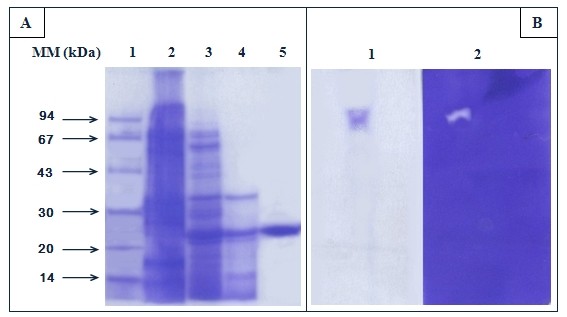
**SDS-PAGE and Native-PAGE of the SCP**. (A) Analysis of purified SCP by SDS-PAGE (13%). Lane 1, molecular mass markers (Pharmacia); lane 2, SCP solution (300 μg) obtained after DEAE-Sephadex chromatography; lane 3, SCP solution (60 μg) obtained after Sephadex G-100 chromatography; lane 4, SCP solution (40 μg) obtained after SP-Sepharose chromatography; lane 5, purified SCP obtained after Sephadex G-50 chromatography (20 μg). The gel was stained with Coomassie blue. (B) Native-PAGE of the purified enzyme, protein stained by Coomassie blue (lane 1); zymogram of the purified chymotrypsin SCP (lane 2).

**- Filtration on Sephadex G-50: **Active fractions eluted from SP-Sepharose column were pooled and loaded onto a gel filtration Sephadex G-50 column (3 × 90 cm) equilibrated with buffer A. Elution of protease was performed with the same buffer at 30 mL/h. The fractions containing the protease activity (eluted at 1.2 void volume) (Figure 1C) (fractions from 75 to 105) were analysed by SDS-PAGE, native-PAGE and by zymography (Figure 2A and 2B).

### SDS-PAGE, native-PAGE and zymography

Analytical polyacrylamide gel electrophoresis of proteins in the presence of SDS-PAGE was performed as described by Laemmli [21]. Native PAGE was performed according to the procedure of Laemmli [21], except that the sample was not heated and SDS and reducing agent were left out. Casein-zymography was performed on native-PAGE according to the method of Garcia-Carreno [19]. Samples were mixed with electrophoresis loading buffer and electrophoresed on a native PAGE. After electrophoresis, the gel was submerged in 100 mL of 1% (w/v) casein in 100 mM Tris-HCl (pH 8.0) for 30 min at 4°C to allow the substrate to penetrate into the gel and then incubated for 45 min at 37°C in enzyme assay buffer (100 mM Tris-HCl pH 8.0) for the development of enzyme activity bands. After washing, the gel was stained with Coomassie Brilliant Blue R-250. Development of a clear zone on the blue background indicated the presence of protease activity.

### Amino acid sequencing

The NH_2_-terminal end of SCP was sequenced by automated Edman's degradation, using an Applied Biosystems Protein Sequencer Procise 492 cLC [22].

## Results and discussion

### Purification of the scorpion protease and general characteristics

A protease was purified from the digestive glands of the scorpion. The purification procedure includes an ammonium sulfate precipitation and chromatographic steps (Table 1). After the last step of Sephadex G-50 chromatography, the purification factor reached 315 with a recovery yield of 45% of the initial protease activity. At this stage, the protease specific activity was found to be 75000 U/mg using casein as substrate at 50°C and at pH 9. The purified enzyme appears as one homogenous band having an apparent molecular mass of 25 kDa (Figure 2A). The molecular mass of scorpion protease was similar to those reported for mammalian and insect chymotrypsin proteinases (22-30 kDa) [23-26]. Furthermore, the zymogram of the purified enzyme under native conditions displayed a protease activity.

**Table 1:** Flow sheet of scorpion chymotrypsin-like protease purification.

Purification step	Total activity* (U) × 10^3^	Protein (mg)	Specific activity (U/mg)	Activity recovery (%)	Purification factor
**Extract of SCP** **(pH 8)**	220 ± 47	925.80 ± 4.68	237.60 ± 8.90	100	1
**DEAE-Sephadex**	179.25 ± 12	203.95 ± 5.90	878.90 ± 25.60	81 ± 5.70	3.70 ± 0.12
**(NH_4_)_2_SO_4_** **Precipitation (75%)**	164.80 ± 8.90	87 ± 3.78	1894.20 ± 98.70	91.90 ± 3.70	7.97 ± 0.43
**Sephadex G-100 chromatography**	103.90 ± 5.40	15.80 ± 0.87	6575.90 ± 578	63 ± 4.06	27.67 ± 0.28
**SP-Sepharose chromatography**	58.23 ± 1.09	0.98 ± 0.23	59418 ± 320	56 ± 0.95	250 ± 2.70
**Sephadex G-50 chromatography**	26.25 ± 1.25	0.35 ± 0.12	75000 ± 540	45 ± 1.05	315.60 ± 6.90

* One unit of protease activity was defined as the amount of enzyme, which liberated 1 μg tyrosine in 1 min at 50°C and at pH 9.

The NH_2_-terminal sequencing of the purified protease allowed the identification of 29 residues: **VEFGRYFRLSEINQFLESLAVTYPEHVI **(Table 2). Using the NH_2_-terminal 29 residues, we run a blast against peptidase families found in the MEROPS database at http://merops.sanger.ac.uk/cgi-bin/blast/submitblast/merops/advanced.

**Table 2:** Alignment of the NH_2_-terminal sequence of scorpion chymotrypsin-like protease with putative peptidases from *Drosophila yakuba *(MER142281), *Drosophila erecta *(MER144486), *Drosophila simulans *(MER136917), *Trichoplusia ni *(MER079404), *Tribolium castaneum *(MER169092) and *Anopheles gambiae *(MER021291) found in the MEROPS Database at http://merops.sanger.ac.uk/cgi-bin/blast/submitblast/merops/advanced.

Potease origin	Access number	NH_2_-Terminal sequence	Identity (%)
***Scorpio maurus ***	This study	**V**E**F**G**RY**F**R**L**SEINQFLE**S**LAVTYP**EH**V**I	-
***Drosophila yakuba***	MER142281	----**RY**YSHE**EINQFIE**D**LAV**K**YP**RR**V**-	60%
***Drosophila erecta***	MER144486	----**RY**YSHE**EINQFIE**D**LA**RK**YP**QR**V**-	56%
***Drosophila simulans***	MER136917	----**RY**YNHE**EINQF**I**E**D**LA**REH**P**----	55%
***Trichoplusia ni***	MER079404	IGFETYY**R**HD**EIN**DY**L**DE**LA**A**TYP**DL**V-**	48%
***Tribolium castaneum***	MER169092	IA**F**DH**Y**L**R**H**SEIN**NY**L**DQ**LA**QN**YP**NI**V**-	48%
***Anopheles gambiae***	MER021291	**V**D**F**EHFWTNA**E**V**N**AY**L**DE**LA**Q**TYP**NL**V-**	40%

Residues in bold indicate the identical amino acids.

The scorpion protease displayed a sequence similarity with putative proteases from various insects such as *Drosophila, Trichoplusia, Tribolium *and *Anopheles *(40-60%) (Table 2). No similarity was found with classical chymotrypsin and trypsin proteases. Similar results were found with scorpion digestive lipase which NH_2_-terminal sequence shares an homology only with insects' putative lipases [13].

Proteases can be classified into various groups based on their mode of action. In order to determine the group of the scorpion protease, the enzyme activity was measured on different synthetic substrates: N-alpha-(p-toluene sulfonyl)-L-arginine methyl ester (TAME) and benzoyl-Arg-*p*-nitroanilide (BAPNA) which are specific of trypsin proteases and N-Succinyl-L-Ala-L-Ala-L-Pro-L-Phe-p-nitroanilide (SAPNA), a specific substrate for chymotrypsin proteases (Table 3). Scorpion protease displayed no activity against trypsin substrates indicating that scorpion protease does not belong to the trypsin group. Interestingly, scorpion protease had a relatively high activity on chymotrypsin substrate (SAPNA). Based on its activity on SAPNA, the scorpion protease can be considered as a chymotrypin-like protease despite the absence of NH_2_-terminal sequence homology with classical chymotrypsins. The purified protease was therefore named Scorpion Chymotrypsin-like Protease (SCP).

**Table 3:** Specific activity of scorpion protease (U/mg) measured on various substrates.

Substrate	TAME^a^	BAPNA^b^	SAPNA^c^	Casein
**Scorpion protease**	< 0.001	0.01 ± 0.002	35 ± 0.65	75000 ± 5400
**Bovine chymotrypsin protease**	nd	nd	280 ± 1.34	197080 ± 8750
**Porcine trypsin protease**	1.3 ± 0.12	4.5 ± 0.4	nd	59125 ± 4800

Bovine chymotrypsin (BCP) and porcine trypsin (PTP) were taken as positive standards for comparative purposes.

Data are expressed as means ± SD of three determinations.

^a^TAME: N alpha-(p-toluene sulfonyl)-L-arginine methyl ester;^b^BAPNA: benzoyl-Arginine-*p*-nitroanilide; ^c^SAPNA: N-Succinyl-L-Ala-L-Ala-L-Pro-L-Phe-p-nitroanilide; nd: not determined.

### Characterization of SCP

#### Effects of temperature on protease activity and stability

**Figure 3:**
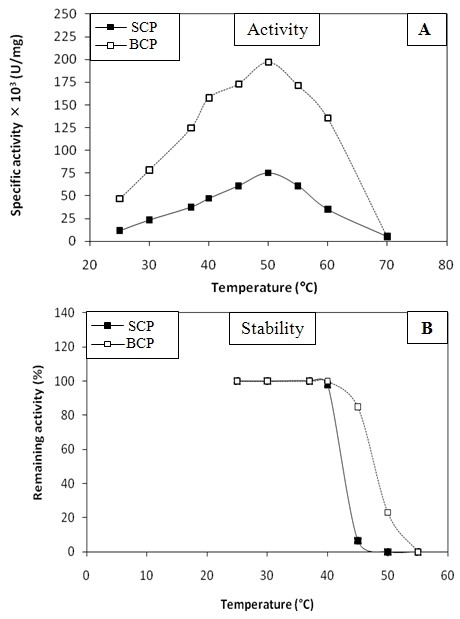
**Effects of temperature**. (A) Effects of temperature on SCP and BCP activities. (B) Effects of temperature on SCP and BCP stabilities. Enzyme activity was tested at various temperatures using casein as substrate at pH 9 for the SCP and at pH 7.5 for the BCP in the presence of 4 mM CaCl_2_. Residual enzyme activity was determined after 15 min in incubation and the initial activity before incubation was taken as 100%.

The effect of the temperature on SCP or bovine chymotrypsin protease (BCP) (taken as a model) activity and stability was checked (Figure 3). The SCP activity increased with the temperature to reach an optimum at 50°C then decreased sharply at higher temperatures (Figure 3A). One can say that SCP is thermoactive which is similar to that of mammalian and fish proteases [6,27,2]. SCP was stable until 40°C and lost its activity after 15 min of incubation at 50°C (Figure 3B). The fact that the enzyme was not stable but fully active at 50°C might explained by the substrate protection effect previously reported for other enzymes [28-30]. Similar results were obtained for BCP (Figure 3A).

#### Effect of pH on protease activity and stability

**Figure 4:**
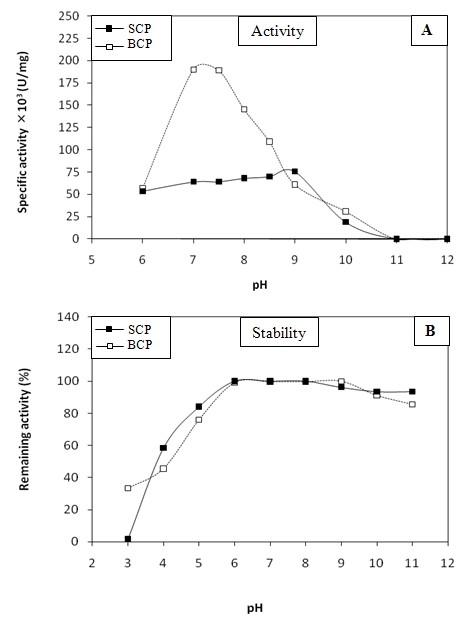
**Effects of pH**. (A) Effects of pH on SCP and BCP activities. (B) Effects of pH on SCP and BCP stabilities. Enzyme activity was tested at various pH using caseine as substrate in the presence of 4 mM CaCl_2 _at 50°C. The pH stability of the protease was determined by incubating the enzyme in different buffers for 1 h at 4°C and the residual activity was measured at pH 9 and at 50°C for the SCP and at pH 7.5 and at 50°C for the BCP. The activity of the enzyme before incubation was taken as 100%.

The effect of pH on the activity and the stability of the purified SCP was also examined and compared to that on BCP. Interestingly, the profile of the activity dependency of the activity on pH was different for SCP and BCP. The SCP displayed a board pH range of activity (from 6 to 9). However, BCP showed a maximal activity at pH 7 (Figure 4A). Both SCP and BCP were stable in a pH range of 6-11 (Figure 4B). The SCP was fully active at pH 9 as was found for chymotrypsin-like proteases from scallop (*Pecten maximus*) [8] and yellow mealworm (*Tenebrio molitor*) [31].

#### Effect of proteinase inhibitors and metal ions

**Figure 5:**
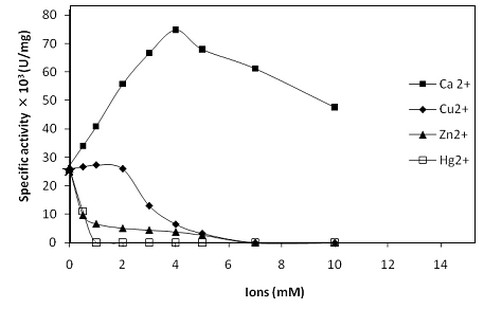
**Effect of ions**. Effect of increasing Ca^2+ ^(black square), Cu^2+ ^(black diamond), Zn^2+ ^(black triangle) and Hg^2+ ^(white square) ions concentrations on the SCP activity using casein as substrate; the star symbol indicates the value of protease activity measured in presence of 10 mM EDTA. SCP activity was measured at 50°C and at pH 9.

The effect of various proteinase inhibitors (Table 4) and metal ions (Figure 5) on the SCP activity was studied. The SCP was strongly inhibited by PMSF, a serine protease inhibitor but not affected by cystein protease inhibitor (DTNB). Furthermore, EDTA which is a metalloprotease inhibitor, showed slight inhibitory effect on SCP (Table 4). These results strongly support that the purified enzyme is a serine protease. Similar inhibition patterns were reported for chymotrypsin-like proteases from scallop [8], lepidopteran insect *Spodoptera exigua *[32] and yellow mealworm [31]. The enzyme incubated with EDTA lost 60% of its initial activity (Figure 5). Only the addition of Ca^2+ ^ions (4 mM) enhanced the SCP activity which reaches its maximal specific activity (75000 U/mg). Nevertheless, Hg^2+^, Zn^2+ ^or Cu^2+ ^inhibited the SCP activity (Figure 5).

**Table 4:** Effect of inhibitors on scorpion chymotrypsin-like protease activity

Inhibitors	Concentration (mM)	Residual activity (%)
**None**		100

**PMSF**	1	18
	5	7

**DTNB**	1	100
	5	100

**EDTA**	1	98
	5	60
	10	40

Enzyme activity measured in the absence of any inhibitor was taken as 100%. The remaining protease activity was measured after incubation of enzyme with each inhibitor at 4°C for 15 min.

## Abbreviations

BCP: bovine chymotrypsin protease; BAPNA: benzoyl-Arg-*p*-nitroanilide; BSA: bovine serum albumine; DMSO: dimethylsulfoxide; DTNB: 5,5'-dithiobis (2-nitrobenzoic acid); EDTA: ethylenediaminetetraacetic acid; FPLC: fast protein liquid chromatography; HPLC: high presure liquid chromatography; kDa: kilodalton; SCP: scorpion chymotrypsin-like protease; SAPNA: N-Succinyl-L-Ala-L-Ala-L-Pro-L-Phe-p-nitroanilide; SDA: scorpion digestive amylase; SDS-PAGE: sodium dodecyl sulfate polyacrylamide gel electrophoresis; TAME: N-alpha-(p-toluene sulfonyl)-L-arginine methyl ester; TEMED: N, N, N', N'-tetramethyl-ethylenediamine; TCA: trichloroacetic acid; PMSF: phenylmethanesulfonyl fluoride; PTP: porcine trypsin protease.

## Competing interests

The authors declare that they have no competing interests.

## Authors' contributions

HL carried out all the studies, analyzed the data and drafted the manuscript. NZ helped with the analysis of the data. NM helped with the discussion of the data and the correction of the manuscript. YG participated in the study design and helped to draft the manuscript. All authors have read and approved the final manuscript.
